# Endocannabinoids, endocannabinoid-like compounds and cortisone in head hair of health care workers as markers of stress and resilience during the early COVID-19 pandemic

**DOI:** 10.1038/s41398-024-02771-9

**Published:** 2024-01-31

**Authors:** Ingeborg Biener, Tonina T. Mueller, Jin Lin, Han Bao, Julius Steffen, Marion Hoerl, Katharina Biere, Sandra Matzel, Tobias Woehrle, Simon König, Annekathrin M. Keiler, Detlef Thieme, Oliver Keppler, Matthias Klein, Tobias Weinberger, Andreas Osterman, Kristina Adorjan, Alexander Choukér

**Affiliations:** 1 Laboratory of Translational Research “Stress and Immunity”, Department of Anesthesiology, LMU University Hospital, LMU Munich, Marchioninistr. 15, 81377 Munich, Germany; 2 Department of Medicine I, LMU University Hospital, LMU Munich, Marchioninistr. 15, 81377 Munich, Germany; 3 Department of Statistics, Ludwig-Maximilians-University Munich (LMU), Ludwigstr. 33, 80539 Munich, Germany; 4 Institute of Doping Analysis und Sports Biochemistry Dresden (IDAS), Dresdner Str. 12, 01731 Kreischa, Germany; 5 Max von Pettenkofer Institute, Virology, National Reference Center for Retroviruses, Faculty of Medicine, Ludwig-Maximilians-University Munich (LMU), Munich, Germany; 6 German Center for Infection Research (DZIF), partner site, Munich, Germany; 7 Emergency Department, LMU University Hospital, LMU Munich, Marchioninistr. 15, 81377 Munich, Germany; 8 Department of Neurology, LMU University Hospital, LMU Munich, Marchioninistr. 15, 81377 Munich, Germany; 9 Department of Psychiatry and Psychotherapy, LMU University Hospital, LMU Munich, Nußbaumstr. 7, 80336 Munich, Germany

**Keywords:** Predictive markers, Human behaviour

## Abstract

The pandemic caused by SARS-CoV-2 impacted health systems globally, creating increased workload and mental stress upon health care workers (HCW). During the first pandemic wave (March to May 2020) in southern Germany, we investigated the impact of stress and the resilience to stress in HCW by measuring changes in hair concentrations of endocannabinoids, endocannabinoid-like compounds and cortisone. HCW (*n* = 178) recruited from multiple occupation and worksites in the LMU-University-Hospital in Munich were interviewed at four interval visits to evaluate mental stress associated with the COVID-19 pandemic. A strand of hair of up to 6 cm in length was sampled once in May 2020, which enabled retrospective individual stress hormone quantifications during that aforementioned time period. Perceived anxiety and impact on mental health were demonstrated to be higher at the beginning of the COVID-19 pandemic and decreased significantly thereafter. Resilience was stable over time, but noted to be lower in women than in men. The concentrations of the endocannabinoid anandamide (AEA) and the structural congeners N-palmitoylethanolamide (PEA), N-oleoylethanolamide (OEA) and N-stearoylethanolamide (SEA) were noted to have decreased significantly over the course of the pandemic. In contrast, the endocannabinoid 2-arachidonoylglycerol (2-AG) levels increased significantly and were found to be higher in nurses, laboratory staff and hospital administration than in physicians. PEA was significantly higher in subjects with a higher resilience but lower in subjects with anxiety. SEA was also noted to be reduced in subjects with anxiety. Nurses had significantly higher cortisone levels than physicians, while female subjects had significant lower cortisone levels than males. Hair samples provided temporal and measurable objective psychophysiological-hormonal information. The hair endocannabinoids/endocannabinoid-like compounds and cortisone correlated to each other and to professions, age and sex quite differentially, relative to specific periods of the COVID-19 pandemic.

## Introduction

The COVID-19 pandemic has challenged global health systems in an unprecedented manner. In Germany, the first recorded COVID-19 patient was diagnosed in Munich, Germany in January 2020 [1]. The first COVID-19 patient was admitted to the LMU University Hospital of Munich at the end of February 2020. The pandemic significantly affected public and civil life in early March 2020, when government quarantine restrictions impacted schools, social interactions and the general economy at large [2]. The health sector was also significantly impacted. Especially health care workers (HCW) were forced to deal with a new and life-threating virus, while the availability of total working personnel declined, resulting in a significant mismatching of resources available to handle the patient load and needs. Additionally, the unknown and/or unavailable treatment possibilities as well as increased limitations in social interactions also affected the anxiety level of HCW [3]. Hence, the impact of COVID-19 and resulting stress appears multifactorial and individually specific.

Assessing and monitoring the individual’s stress response and resilience in a standardized fashion is typically performed using self-reported validated questionnaires. However, self-reported stress often differs from physiological responses and objective measurements [4, 5]. Therefore, monitoring biological stress response markers is useful to complement self-assessment by identifying and validating suitable and evolving biomarkers of chronic stress and resilience.

Cortisol is known to be one of the key biomarkers of the biological stress response. In this process, the activation of the HPA axis [6] and the sympathetic nervous system culminate in the acute and short term release of glucocorticoids (i.e. cortisol) and catecholamines from the adrenal cortex and medulla [7], respectively. As circadian cortisol fluctuations occur in saliva, urine or blood, they do not provide a practical way to validate any long-term baseline changes [8]. The physiological slow uptake of cortisol within hair better reflects these changes over a longer interval [9]. Since hair cortisone and hair cortisol correlate strongly [10], the biologically inactive cortisone, with its higher concentrations in hair, is a very suitable and reliable surrogate marker of stress in healthy individuals. Given the average hair growth rate of 1 cm per month [8], it is possible to perform a retrospective analysis over several months, including for the time period before the COVID-19 pandemic. However, this analysis is limited by the steadily decreasing corticosteroids concentrations noted after initial incorporation into hair segments [11, 12]. Hair chemical treatments, natural sunlight and artificial UV-radiation result in corticosteroid degradation and cause a time-dependent decrease in the cortisol concentration found in hair samples [8, 13]. While the time-dependent decrease in corticosteroid concentrations make longitudinal analysis impossible, cross-sectional comparisons of different individuals from similar time points in hair production remain relevant [14].

In addition to the above mentioned systems, the endocannabinoid system (ECS) is an important endogenous stress response system [7, 15–17]. It is critical to maintaining body hemostasis through balancing and regulating multiple vegetative physiological functions. These processes are regulated by the endocannabinoids anandamide (AEA) and 2-arachidonoylglycerol (2-AG), and their structural congeners N-acylethanolamines (NAEs). The most studied NAEs are N-palmitoylethanolamide (PEA), N-oleoylethanolamide (OEA) and N-stearoylethanolamide (SEA). The endocannabinoids and the endocannabinoid-like lipid mediators are involved in a wide range of biological pathways, including regulating appetite, nutrient metabolism, energy balance and inflammation [18]. Endocannabinoids can also be measured in hair without the significant molecular degradation found with corticoids [11]. Thus, endocannabinoid analyses in hair specimens may enable inductive monitoring of stress responses, specifically AEA and 2-AG, which are associated with stress-induced activation of the HPA axis [19].

Multiple studies have investigated stress in HCW utilizing questionnaires, alone or in combination with salivary or hair cortisol/cortisone concentrations, during the COVID-19 pandemic [5, 20–25]. Because of these important physiological roles of endocannabinoids and endocannabinoid-like compounds in the stress response, in this study we attempted to verify their role relative to the stress impact of the pandemic on HCW. We specifically hypothesized that stress and the resilience to stress in HCW of different professions, sex, and age resulted in temporal measurable concentration changes of the endocannabinoids and endocannabinoids-like compounds and cortisone found in hair segments.

## Materials and Methods

### Study design and population

**Fig. 1:**
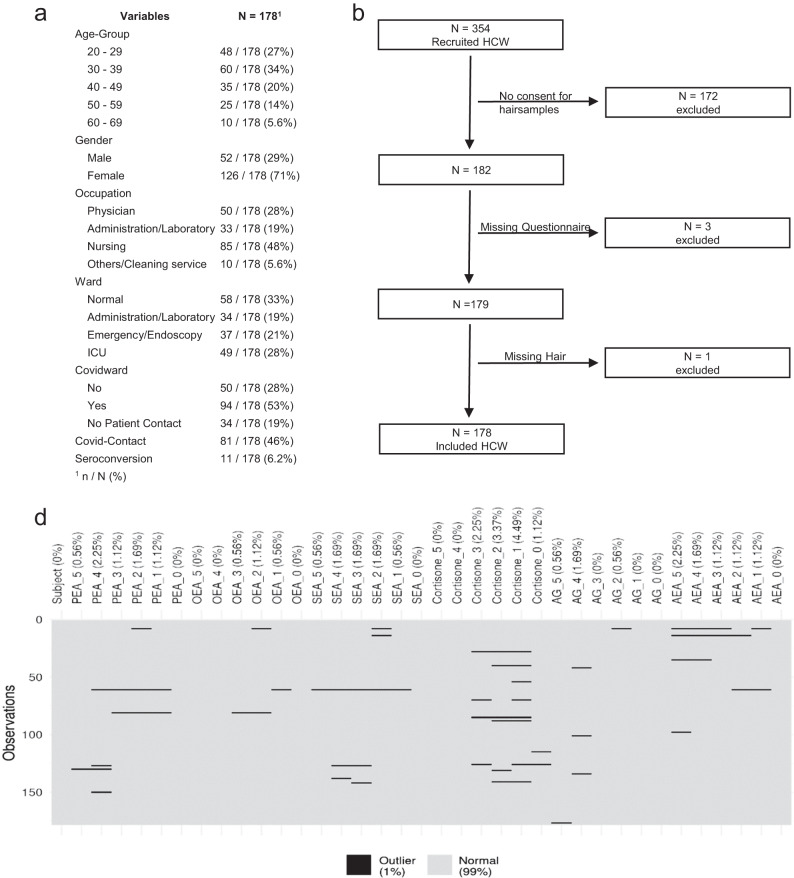

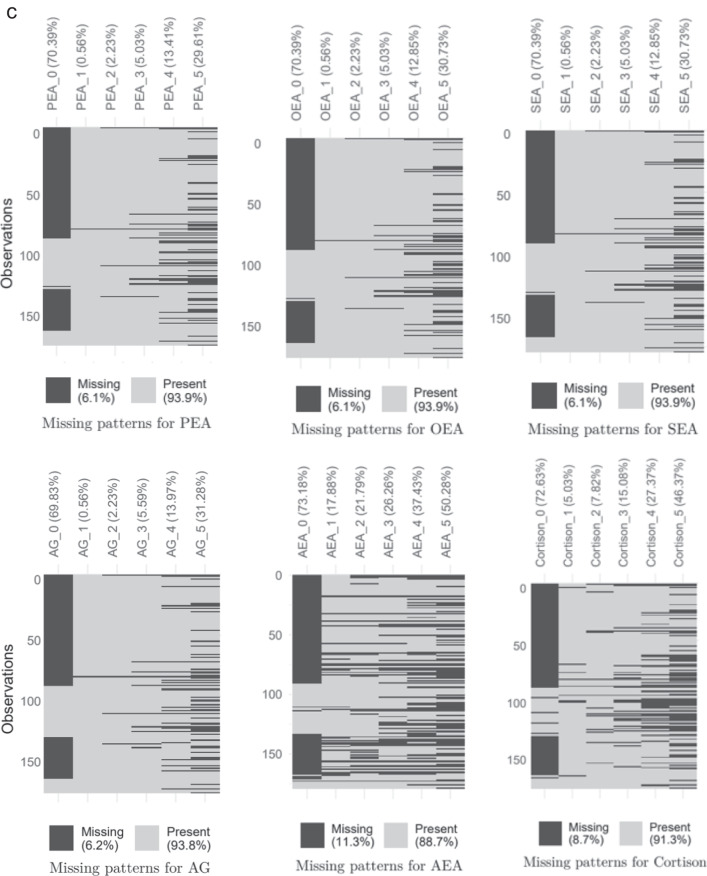
Synopsis of subjects. **a** Categorical variables for all subjects. **b** Study flow chart: illustrating the recruiting process: 354 HCW who were mostly recruited for the prospective longitudinal serosurvey (Weinberger, T., et al. [27]) were asked to participate in our study. 182 subjects gave their approval, while 3 subjects were excluded because of missing questionnaire-data and one because of missing hair, hereby resulting in a total number of 178 subjects included to the analyses. **c** Missing hair data for all hormones at every time point. Because of hair length, hair cutting time and characteristics of the hormones differ the number of missing data for each hormone and for each time-point. **d** Outliers of the hormone data: extreme values were removed, when the mean value was ± 3 times the standard deviation of the log-transformed value; AEA: 12 outliers in 5 subjects, PEA: 12 outliers in 6 subjects, OEA: 11 outliers in 3 subjects, SEA: 11 outliers in 6 subjects, 2-AG: 5 outliers in 5 subjects, cortisone: 3 outliers in 3 subjects.

This study was performed at the LMU University Hospital in Munich, Germany. It is among the largest hospitals in Germany, treating more than half a million patients each year under the care of 11000 staff members from more than 100 departments [26]. Following an internal announcement, we recruited staff members (*n* = 354) from different occupations and worksites, who mostly participated in a study targeting the seroconversion rates during the first wave of the COVID-19 pandemic [27]. After obtaining Investigational Review Board (IRB) approval, 182 subjects providing informed consent were additionally enrolled for hair sampling studies. Four subjects were subsequently excluded due to missing data (Fig. 1b).

**Fig. 2:**
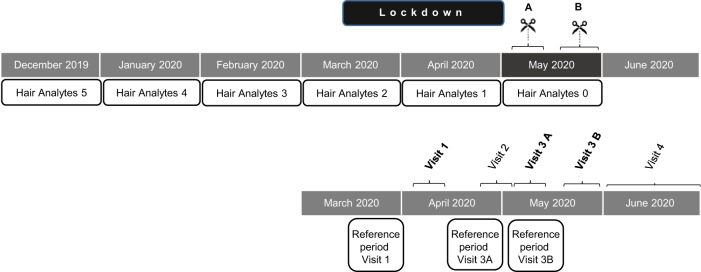
Timetable of the study. Study protocol time points for the visits and the corresponding hair segments of the hair strain mirroring the past months accordingly and the corresponding questionnaires. Upper part (hairsampling): The hair sample was taken in May either in the time frame A or in the time frame B (A: May 6 to 15, 2020; B: May 18 to 29, 2020). The hair strain was cut in 1 cm segments, corresponding to the respective month. Lower part (questionnaire): Each subject was visited four times and questionnaires were filled out asking for a retrospective period of two weeks (reference period). At visit 1 and visit 3 (split into two periods A and B) questionnaires were corresponding to the hair analyt period 0 and 1 or 2. At visit 2 and 4 no correlation occurred with hair sampling.

The subjects were asked to fill in a paper-based questionnaire evaluating mental stress and resilience, as well as provide hair strand sampling at the following time points: between April 6 to 16, 2020 (Visit 1), between April 23 to May 12, 2020 (Visit 2). Visit 3 occurred between May 6 to 29, 2020 (with one subject exception occurring June 5, 2020). Since hair grows at about 1 cm per month, we assigned the hair samples that were taken until May 15, 2020, to the month of April and the hair samples that were taken after May 18, 2020 to the month of May in order to limit the error in allocating the hair strand to its corresponding growth period. Visit 4 was between June 1 to July 1, 2020 (Fig. 2).

All collected data was pseudonymized and additionally double pseudonymized when biosamples were transferred to laboratory staff. The study was approved by the IRB of the Medical Faculty of the LMU (No: 20-247) [27].

### Questionnaire

A paper-based questionnaire was applied for the Visits 1, 2, 3 and 4 (Supplement 1). To achieve the highest possible standardization, the questions were extracted from the COVID-19 Pandemic Mental Health Questionnaire (CoPaQ) [28]. Moreover, the validated brief resilience scale (BRS) was applied for the Visits 2–4 [29, 30]. Each questionnaire consists of three blocks of questions that relate specifically to the period of 2 weeks before the visit. In the first section, we asked about anxiety related to the COVID-19 pandemic (question-set I), adhering to the new COVID-19 rules (question-set II) and how the pandemic has changed the psychological health of each participant (question-set III). Each question was rated by the subject on a five-point scale where the highest number `4´ corresponds to “totally agree” with the statement, to the lowest number `0´, corresponding to “do not agree” with the statement. Beside using that five-point self-assessment scale we opted also for some supplemental analyses to categorize the questionnaire results into “Low Stress” and “Stress” using the sum of participant’s scores across each question set similar to categorization of the resilience scale. This allocation in categories is called factorization.

Numeric scores in each set of questions provided sums and mean scores for analysis, where indicated.

Based on the maximal point sum counted in question sets I, II and III, an allocation (factorization) occurred into two groups, “Low Stress” and “Stress”, as follows: Sums of rating of the question-sets I and II: ≤ 6 points “Low Stress”, >7 points “Stress” and sums of rating of the question question-set III: ≤8 ”Low Stress”, >9 “Stress”. During the Visit 2, 3 and 4 we complemented the stress questionnaire measurements with the state of the art brief resilience scale (BRS) (question-set IV) [29, 30]. The scores for each of the six questions range between values of 1 to 5. The resulting resilience score sum was calculated and divided by the number of questions (six). As recommended by Smith et al. [31], an allocation (factorization) of the BRS-score occurred into three groups: a BRS-score below 3 attributed to the subject a “low resilience”, a BRS-score between 3 and 4.3 attributed a “normal resilience”, and a BRS-score of 4.3 and above a “high resilience”.

### LC-MS/MS measurement for hair analysis

A single strand of hair was cut with scissors from an area behind the ear as close as possible to the scalp with a length of up to 6 cm to quantify the analytes (Fig. 1c). The hair strand was then cut into 1 cm segments, identified as proximal to distal interval segments and then ground using a hair mill. 1 ml MeOH and internal standards (see below) were subsequently added, and the samples were homogenized using a Fast-Prep 24 (MP Biomedicals™, Thermo Fisher Scientific), and incubated in the ultrasonic bath (Bandelin Sonorex Super RK 510H) at 50 °C for 12 h and subsequently centrifuged (Eppendorf Centrifuge). 800 µl of the MeOH supernatant were mixed with 20 µl Ethylenglycol (J.T.Baker, Deventer, Netherlands) and subjected to the vacuum evaporator (Christ, RVC 2-25CO plus). Solvents A2/ B2 (40µl1:4 v/v) (see below) were added to the evaporated samples, mixed and transferred to the HPLC-vials. After this preparation, the samples underwent a second pseudonymization before being analyzed at the co-authors´ IDAS laboratory (Kreischa, Germany). The quantifications of the hair analytes included the use of an Agilent 1290 Infinity liquid chromatography LC system (Agilent Technologies, Böblingen, Germany) and a Triple TOF 6600 mass spectrometer (AB Sciex, Darmstadt, Germany), respectively. Chromatographic separation was carried out using an Eclipse XDB-C8 column (2.1 x 100 mm, 3.5 µm; Agilent Technologies, Böblingen, Germany) with the following gradient: 0 min 10% B, 1 min 10% B, 10 min 100% B, 12:50 min 100% B and 13 min 10% B, with A (ACN/H2O, 5/95 v/v containing 2 mmol/L ammonium acetate and 0.1% acetic acid) and B (ACN/H2O, 95/5 v/v containing 2 mmol/L ammonium acetate and 0.1% acetic acid) (ACN: gradient grade, J.T. Baker, Deverner, Netherlands, H2O: gradient grade, Fisher scientific, Schwerte, Germany, acetic acid: Th. Geyer, Renningen, Germany, Ammonium Acetate: Merck, Darmstadt, Germany). Flow rate was set at 300 µl/min, the injection volume was 10 μl.

Endocannabinoids and endocannabinoid-like compounds (AEA, 2-AG, OEA, PEA, SEA) and cortisone (E) were quantified in high sensitivity HR-MS-MS mode with positive ionization. The following fragmentation reactions were monitored and used for quantitation in high sensitivity HR-MS-MS mode with positive ionization (Supplement 2). Cortisone was used as analyte because it was shown to correlate highly with the active compound cortisol and is more reliable when quantifying it from hair than cortisol [10]. For solvent calibration the analytes were spiked into matrix free methanol containing internal standards (d4-AEA 0.1 ng, d5-2AG 10 ng, 33 ng d4-PEA, and d3-SEA, d4-OEA 100 ng each Cayman Chemical, Ann Arbor, USA; 0.5 ng d8-cortisone (Sigma Aldrich, Merck, Darmstadt, Germany)) and processed similarly to the hair samples. A standard calibration curve was obtained in the following concentration ranges AEA (0–60 pg/mg), 2-AG (0–2000 pg/mg), SEA, PEA, OEA (each 0–50.000 pg/mg) and E (0–200 pg/mg). The analysis was achieved using Analyst TF® 1.71 and MultiQuant 3.0 software (Sciex, Darmstadt, Germany).

### Statistical analysis

The statistical analyses were conducted independently by LMU University statisticians, who were not involved in the study design, its execution, questionnaire acquisition or biosample analyses. Statistics were performed using R Statistical Software (R version 4.2.0; The R Foundation, Vienna, Austria). Chi-squared test was used to verify the correlation between categorical variables in the hormone data. Through Fisher’s exact test, variables were classified into four major categories based on the correlation’s relationship: basic features (Age, Gender), occupational characteristics (Occupation), hair-related features (Natural Hair-Color, Colored, Tinted), and COVID-related features (COVID-19 Contact, Seroconversion). The variables are inter-category independent, while there exists some degree of correlation among variables intra-category.

Descriptive data was presented as frequency tables for the demographic variables, as well as line plots and boxplots for hormones values. Linear mixed models were performed with the lme4 package in R to score for fear and resilience, based on the psychological questionnaire data and were used for the longitudinal analysis of all hormone levels as log-transformed values in the merged data (*n* = 178). Interclass correlation coefficients (ICC) were applied to address the correction of multiple testing in generalized linear mixed models (GLMM).

Under logarithmization, this threshold became reasonable, because of the more symmetric data distribution and stabilized variance. Due to the presence of extreme outliers in the dataset, applying conventional outlier detection criteria, such as z-score or standard deviation, would significantly compromise the effectiveness of downstream analysis. Therefore, aiming to mitigate the impact of outliers, while retaining a certain amount of valid data, a criterion based on the mean ± 3 standard deviations was applied. This choice ensures that, even in the context of a small sample size, more than 90% of the data remains available for downstream analysis (Fig. 1d). Linear regression was used for cross-sectional analysis of all hormone values at Visit 1 and Visit 3.

## Results

### Cohort details

Among all subjects enrolled, 178 were deemed valid for study inclusion and further statistical analyses (Fig. 1b). The study population encompasses an age range from 20 to 69 years, with more than 70% being female staff. As per profession, the cohort of nurses represented almost 50% of the total number, while doctors constituted 28% and administrative/ laboratory staff almost 20% of all subjects. The remaining employees (i.e. cleaning staff etc.) represented a very small portion of about 5%. The locations of different workplaces were more evenly distributed. 20–30% of the staff worked either on normal wards, in the administration and laboratory, in the emergency room or endoscopy suite, or on the intensive care units. Some of these workplaces involved patients with or without COVID- 19, while some were without patient contact (Fig. 1a).

### Questionnaires on stress and anxiety (set I), adhering the new COVID-19 rules (set II), psychological health (set III) and resilience (set IV)

**Fig. 3:**
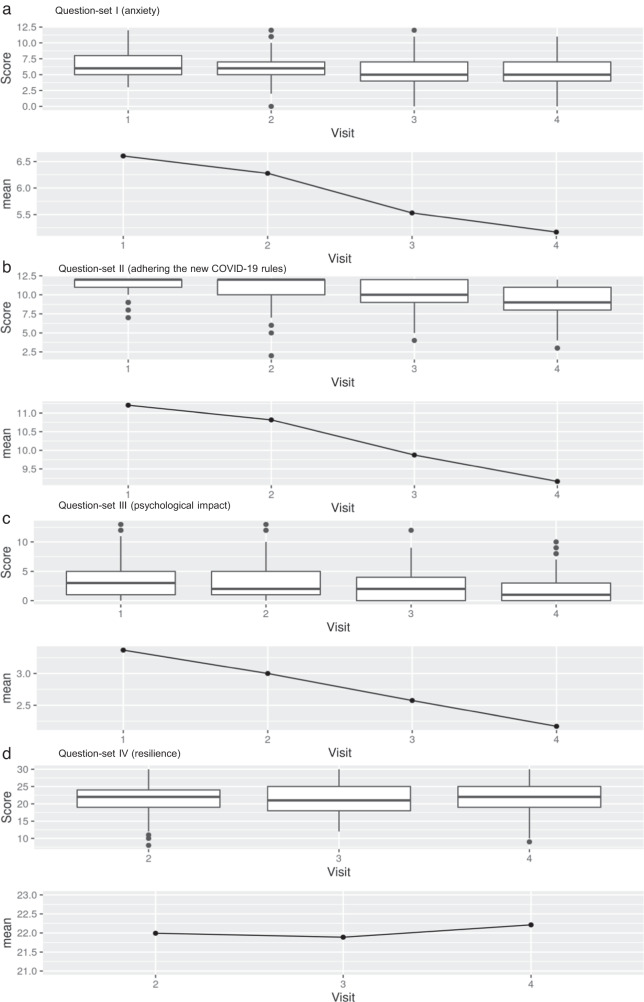
Boxplot of Question-sets I-IV. In the Question-sets I-III (**a**, **b**, **c**) a significant decrease of anxiety (**a**), adhering the new COVID-19 rules (**b**) and psychological impact (**c**) over the time period was observed. The resilience (Question-set IV, **d**) remained stable.

The subjective perceived anxiety (question-set I) and the impact on the psychological health (question-set III) were rated higher at the beginning of the COVID-19 pandemic (Visit 1) and decreased significantly over time (question-set I: −0.68, *p* < 0.001; question-set III: −0.39, *p* < 0.001) (Fig. 3a, c, Supplement 3). Although there was only a small difference, there was a change in results from question-set I from stressed to low stressed, between the first two visits and the subsequent final two visits. Adherence to the new COVID-19 rules (question-set II) became significantly smaller (−0.73, *p* < 0.001) in the later successive visits (Fig. 3b).

Resilience remained repeatedly stable and with a wide variance between the subjects (variance of random intercept: 12.79) at all three time points; there was no significant change (0.04, *p* = 0.748) (Fig. 3d).

The age groups 40 to 49 and 50 to 59 years showed significantly higher stress levels (question-set I), than the age group 20 to 29 years (40–49: 1.25, *p* = 0.010; 50–59: 1.51, *p* = 0.005). There was no significant difference between working on the normal wards or in the intensive care unit (0.21, *p* = 0.616) nor between working on a COVID-19- versus non-COVID-19 ward (−0.23, *p* = 0.544). There was a trend towards higher stress levels for the occupation administration/laboratory, when compared to physicians (0.90, *p* = 0.080). Such a trend towards higher stress levels was observed in those personnel working without patient contact (e.g. hospital administration/laboratory (Supplement 3 (3a)) as compared to ward staff (0.91, *p* = 0.065).

Nurses had significant lower values indicating the impact from adhering to the new COVID-19 rules (question-set II) (−0.57, *p* = 0.044). Their compliance in following recommendations and in limiting social contacts was lower when compared to physicians. The age group 60–69 years, which is a smaller group with 10 subjects, reported a higher impact adhering to the new COVID-19 rules, than the age group 20–29 (1.51, *p* = 0.004) (Supplement 3 (3b)).

Women showed a higher negative impact on their psychological health (question-set III) than men. Also the age group 50–59 years reported a higher negative impact than the age group 20–29 years (1.58, *p* = 0.004). In the endoscopy/emergency units, the impact was lower than on the normal wards (−0.91, *p* = 0.042). As in question-set I, there was no significance, but merely a trend (0.83, *p* = 0.107) for a higher impact on psychological health for the occupation administration/laboratory (Supplement 3 (3c)).

The resilience of women was lower than that of men (−2.03, *p* = 0.003), as was also found for staff working at the administration/laboratory than for physicians (−2.08, *p* = 0.022). A non-significant trend towards lower resilience was found in the age group 50–59 years, than in the age group 20–29 years (−1.44, *p* = 0.131) (Supplement 3 (3d)).

### Hair endocannabinoids and endocannabinoid-like compounds

**Fig. 4:**
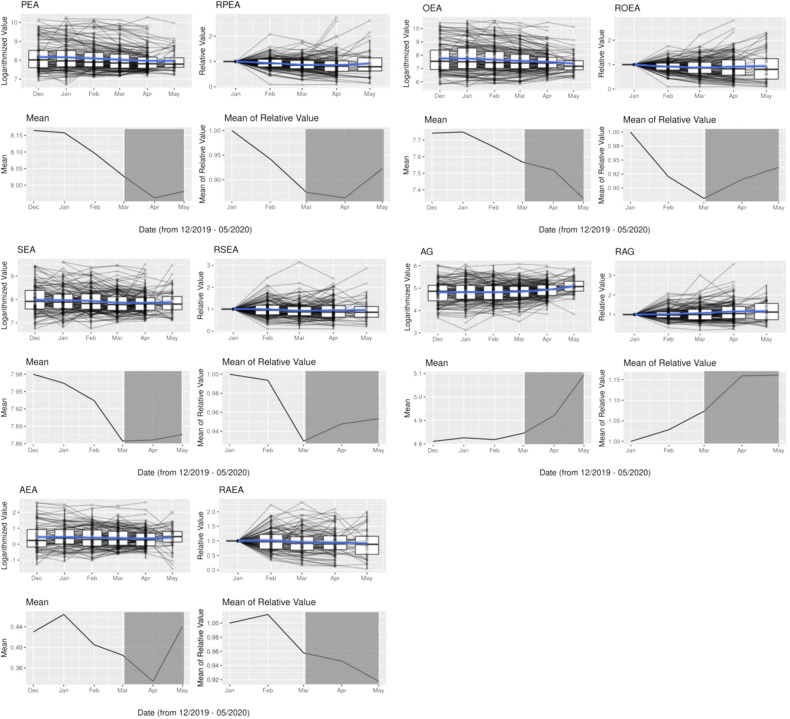
Line_plots of endocannabinoids. Line_plots of endocannabinoids/ endocannabinoid- like-compounds for the absolute logarithmized numbers (without outliers) and for the relative values with January as reference. The lockdown period is marked dark grey. There was a significant decrease (*p* ≤ 0.001) for PEA, OEA, SEA and AEA and a significant increase (*p* < 0.001) for 2-AG over time (see also supplement 5 for the statistical calculation).

Over the observation period of several months, the targeted endocannabinoids and endocannabinoid-like compounds were quantifiable in the hair segments in considerable concentrations. The overall changes in the concentrations over time were not large, but described a differential pattern for the endocannabinoids/ endocannabinoid-like compounds, PEA, OEA, SEA and AEA. These decreased over time significantly (*p* ≤ 0.001), also as expressed in relative values compared to the pre-pandemic time point in January. The mean value of PEA (without outliers) decreased from 5343,45 pg/mg hair in January to 3341,46 pg/mg in May, the mean value of OEA from 4811,12 pg/mg to 1860,08 pg/mg, the mean value of SEA from 3499,72 pg/mg to 2853,30 pg/mg and the mean value of AEA from 2,87 pg/mg to 2,09 pg/mg. In contrast, 2-AG increased significantly (*p* < 0.001; 145,97 pg/mg in January and 176,20 pg/mg in May) (Fig. 4).

The linear model analyses at Visit 3 showed the PEA concentrations to be significantly higher in subjects with a higher resilience (0.02, *p* = 0.046) and lower in subjects with high anxiety at Visit 3 (−0.08, *p* < 0,001). The latter was also seen for SEA, but a bit less pronounced (−0.05, *p* = 0.028) (Table 1).

**Table 1:** Linear Model of endocannabinoids at visit 3.

	PEA		OEA		SEA		2-AG		AEA	
*Predictors*	*Estimates*	*p*	*Estimates*	*p*	*Estimates*	*p*	*Estimates*	*p*	*Estimates*	*p*
(Intercept)	7.58	**<0.001**	7.03	**<0.001**	7.65	**<0.001**	5.20	**<0.001**	0.03	0.956
	(6.87–8.29)		(6.12–7.94)		(7.01–8.30)		(4.74–5.66)		(−0.99–1.04)	
Age−Group:	*Reference*		*Reference*		*Reference*		*Reference*		*Reference*	
20 to 29										
Age-Group:	0.08	0.458	0.08	0.567	0.06	0.559	−0.01	0.865	−0.04	0.806
30 to 39	(−0.13–0.29)		(−0.19–0.35)		(−0.13–0.24)		(−0.15–0.12)		(−0.34–0.27)	
Age−Group:	0.03	0.799	0.06	0.709	−0.01	0.933	0.04	0.613	0.04	0.816
40 to 49	(−0.21–0.27)		(−0.25–0.36)		(−0.22–0.20)		(−0.11–0.19)		(−0.32–0.41)	
Age-Group:	0.15	0.284	0.19	0.299	0.08	0.536	0.06	0.543	−0.01	0.957
50 to 59	(−0.13–0.43)		(−0.17–0.55)		(−0.18–0.34)		(−0.13–0.24)		(−0.41–0.39)	
Age-Group:	−0.18	0.383	0.10	0.713	−0.06	0.759	0.28	**0.032**	0.11	0.751
60 to 69	(−0.57–0.22)		(−0.42–0.61)		(−0.42–0.30)		(0.03–0.54)		(−0.58–0.80)	
Gender: Male	*Reference*		*Reference*		*Reference*		*Reference*		*Reference*	
Gender: Female	0.15	0.130	0.04	0.771	0.01	0.869	−0.08	0.217	0.00	0.980
	(−0.05–0.35)		(−0.22–0.29)		(−0.16–0.19)		(−0.21–0.05)		(−0.31–0.32)	
Occupation: Physician	*Reference*		*Reference*		*Reference*		*Reference*		*Reference*	
Occupation:	0.21	0.102	0.22	0.188	0.25	**0.037**	0.27	**0.002**	−0.10	0.621
Administration/ Lab.	(−0.04–0.47)		(−0.11–0.56)		(0.01–0.48)		(0.10–0.44)		(−0.48–0.29)	
Occupation: Nursing	−0.01	0.931	0.00	0.978	0.02	0.809	0.18	**0.008**	0.20	0.199
	(−0.22–0.20)		(−0.26–0.27)		(−0.16–0.21)		(0.05–0.32)		(−0.11–0.52)	
Occupation:	−0.02	0.936	0.07	0.777	0.11	0.551	0.18	0.165	0.25	0.415
Others/Cleaning	(−0.42–0.39)		(−0.44–0.59)		(−0.25–0.47)		(−0.07–0.43)		(−0.35–0.85)	
CovidContact: No	*Reference*		*Reference*		*Reference*		*Reference*		*Reference*	
CovidContact: Yes	0.10	0.264	0.07	0.532	0.15	0.064	0.11	0.056	−0.08	0.558
	(−0.08–0.28)		(−0.16–0.30)		(−0.01–0.31)		(−0.00–0.23)		(−0.36–0.19)	
Visit 3 Stress / Anxiety	−0.08	**<0.001**	−0.04	0.216	−0.05	**0.028**	−0.04	**0.012**	−0.04	0.212
	(−0.13– −0.04)		(−0.09–0.02)		(−0.09– −0.01)		(−0.07– −0.01)		(−0.11–0.02)	
Visit 3 Adhering rules	0.01	0.749	−0.01	0.679	0.01	0.530	−0.01	0.454	0.04	0.254
	(−0.04–0.05)		(−0.07–0.04)		(−0.03–0.05)		(−0.04–0.02)		(−0.03–0.10)	
Visit 3 Effects of the Pandemic	0.04	0.060	0.02	0.400	0.00	0.890	0.01	0.225	0.03	0.275
	(−0.00–0.07)		(−0.03–0.07)		(−0.03–0.04)		(−0.01–0.04)		(−0.02–0.09)	
Visit 3 Resilience	0.02	**0.046**	0.02	0.076	0.01	0.365	−0.01	0.355	0.00	0.999
	(0.00–0.04)		(−0.00–0.05)		(−0.01–0.03)		(−0.02–0.01)		(−0.03–0.03)	
Observations	156		155		155		156		126	
R^2^ / R^2^ adjusted	0.178 / 0.103		0.064 / −0.022		0.106 / 0.024		0.195 / 0.121		0.071 / −0.037	

Linear model at Visit 3: PEA is higher with higher resilience and lower with subjects with stress. SEA is higher with subjects working in the administration/laboratory and lower with stress. 2-AG is higher in nurses and workers from administration/laboratory and in the age group 60–69 at visit 3.

Bold values identify statistical significance (*p* < 0.05).

SEA concentrations were higher in subjects working in the administration/laboratory (0.25, *p* = 0.037), a tendency also seen in PEA concentrations (0.21, *p* = 0.102) (Table 1). The statistical significance for SEA was weak and seen only in the original scores. When the values were further allocated/factorized (as explained in methods) into groups (e.g. stressed, not stressed; low, normal or high resilience) this statistical significance persisted for PEA, but not for SEA (Supplement 4).

More pronounced changes were observed for 2-AG in the staff. Nurses and staff who worked in the laboratory or administration had higher 2-AG than physicians throughout the whole observation period. This was evident in the linear model analyses combined with the questionnaire and in the mixed models without questionnaire (Supplement 5). At Visit 3, the age group 60–69 years had in comparison to the age group 20–29 years higher 2-AG-levels (0.28, *p* = 0.032) (Table 1).

No significance was observed for OEA and AEA in the study cohort (Table 1).

#### Hair cortisone

As cortisone is known to be degraded as a function of “hair-age” [11] a longitudinal analysis was not possible. Therefore, only cross-sectional comparisons at fixed time points could be conducted. Linear model for cortisone at Visit 1 (during the first wave of the pandemic in Germany) and at Visit 3 still showed significantly higher cortisone concentrations in nurses than physicians. Cortisone was significantly higher at Visit 1 and 3 in nurses than in physicians (*p* = 0.016/ *p* = 0.020), respectively (Table 2). There was a strong statistical significance between occupating “Nursing” and high cortisone levels in all further analyses, including when the Questionnaire score was factored into the different groups (Supplement 6). A higher cortisone level was seen in the age range of 40–49 years at Visit 3 for factorized stress. A trend towards lower cortisone levels was seen in females compared to males at Visit 1 (n.s., *p* = 0.193), but was significantly lower in females than in males at Visit 3 (*p* = 0.012) (Table 2).

**Table 2:** Linear model for Cortisone.

2a	Linear model of Cortisone at visit 1	
*Predictors*	*Estimates*	*p*
(Intercept)	36.97	**0.022**
	(5.33–68.62)	
AgeGroup: Age 20 to 29	*Reference*	
AgeGroup: Age 30 to 39	−4.63	0.203
	(−11.79–2.54)	
AgeGroup: Age 40 to 49	3.55	0.394
	(−4.68–11.78)	
AgeGroup: Age 50 to 59	−1.18	0.789
	(−9.85–7.50)	
AgeGroup: Age 60 to 69	−1.56	0.829
	(−15.87–12.75)	
Gender: Male	*Reference*	
Gender: Female	−4.67	0.193
	(−11.75–2.40)	
Occupation: Physician	*Reference*	
Occupation:	1.60	0.727
Administration/Virology	(−7.45–10.65)	
Occupation: Nursing	8.87	**0.016**
	(1.67–16.07)	
Occupation:	−3.70	0.651
Others/Cleaning service	(−19.84–12.45)	
CovidContact: No	*Reference*	
CovidContact: Yes	−4.51	0.146
	(−10.63–1.60)	
Seroconversion: No	*Reference*	
Seroconversion: Yes	−1.43	0.874
	(−19.28–16.41)	
Block I: Stress / Anxiety	0.99	0.226
	(−0.62–2.60)	
Block II: Behavior Change	−1.13	0.359
	(−3.56–1.30)	
Block III: Effects of the	−0.69	0.186
Pandemic	(−1.72–0.34)	
Block IV: Dealing with	0.06	0.863
the Pandemic	(−0.65–0.78)	
Observations	116	
R^2^ / R^2^ adjusted	0.158 / 0.042	

2b	Linear model of Cortisone at visit 3	
*Predictors*	*Estimates*	*p*
(Intercept)	45.97	**0.001**
	(19.55–72.39)	
AgeGroup: Age 20 to 29	*Reference*	
AgeGroup: Age 30 to 39	−2.06	0.602
	(−9.86–5.74)	
AgeGroup: Age 40 to 49	7.84	0.076
	(−0.83–16.52)	
AgeGroup: Age 50 to 59	3.51	0.499
	(−6.71–13.73)	
AgeGroup: Age 60 to 69	1.55	0.849
	(−14.46–17.56)	
Gender: Male	*Reference*	
Gender: Female	−9.58	**0.012**
	(−16.98–−2.18)	
Occupation: Physician	*Reference*	
Occupation:	6.05	0.223
Administration/Virology	(−3.72–15.82)	
Occupation: Nursing	9.38	**0.020**
	(1.52–17.23)	
Occupation:	5.14	0.530
Others/Cleaning service	(−10.99–21.28)	
CovidContact: No	*Reference*	
CovidContact: Yes	1.10	0.742
	(−5.46–7.66)	
Seroconversion: No	*Reference*	
Seroconversion: Yes	3.45	0.617
	(−10.18–17.09)	
Block I: Stress / Anxiety	0.20	0.808
	(−1.43–1.83)	
Block II: Behavior Change	−0.09	0.909
	(−1.67–1.49)	
Block III: Effects of the	0.51	0.450
Pandemic	(−0.83–1.85)	
Block IV: Dealing with	−0.49	0.214
the Pandemic	(−1.26–0.28)	
Observations	148	
R^2^ / R^2^ adjusted	0.121 / 0.029	

Linear model for Cortisone at visit 1 (2a) and visit 3 (2b); significant higher cortisone was seen in the occupation group nurses in comparison to the group physician. At Visit 3 also a significantly lower cortisone was seen in female compared to male.

Bold values identify statistical significance (*p* < 0.05).

## Discussion

The COVID-19 pandemic provided a great challenge to society and in particular health care workers (HCW). Our data suggests a measurable effect of the multi-faceted stress impact at the beginning of the pandemic on hospital staff. Using both subjective surveys (including stress and resilience questionnaires) and an objective analysis of different stress hormones in hair samples, we infer previously unknown factors impacting psychological health of HCW. As we hypothesized, the individual mean stress level in the cohort was higher at the beginning of the pandemic than at the end of the first wave, as was confirmed in other studies in comparable settings [32]. Croghan et al. [21] reported a higher stress level and a lower resilience in nurses than other job categories, including medical doctors, physician assistants and nurse practitioners. In our study, we did not identify a significant difference between the subjective stress-level among the different occupation groups. However, there was a non-statistically significant trend of higher stress levels among the administration and laboratory staff in contrast to physicians, while differences in age and sex were statistically significant. Even when nurses did not report higher stress levels in the questionnaire, which also could be attributed to a difference between self-reporting and actual feeling [4, 5, 33], we saw significantly higher biological responses as determined by the cortisone levels in hair specimens of nurses when compared to physicians. Rajcani et al. [24] also quantified higher cortisol levels in nurses during the pandemic in comparison to prior baseline. However, as cortisol in hair is not stable over a longer time period [11, 12], it is likely that the higher cortisol level observed in their study during the pandemic period occurred because these hair strands were evaluated closer to the hair root. Nevertheless, Rajcani [24] could also show higher cortisol levels in nurses working in settings with higher COVID-19 contact risk than compared to nurses working in lower risk environments. While an adjusted secretion of glucocorticoids in acute stress is positive and important for the individual, abnormal glucocorticoid release due to chronic stress can cause multiple stress-related diseases (e.g. post-traumatic stress disorder) [34]. Accordingly, the higher hair cortisol levels in individuals that had been obtained six years before the COVID-19 pandemic were seen in the same individuals to be associated with clinically significant depression during the pandemic [35].

Moreover, in our survey, female HCWs reported a higher negative impact on their mental health and a lower resilience, as defined by the American psychological Association as “the process of adapting well in the face of adversity, trauma, tragedy, threats or significant sources of stress” [36]. This relationship was also shown in a study performed in more than 4000 subjects of the general public during COVID-19 confinement in Spain [37]. In contrast to these studies, a short observational study covering a period of one week during lockdown showed a higher stress response in men [38]. In some animal and human studies, resilience was associated with fast activation and fast termination of the stress response of the HPA axis [39, 40]. In order to measure this individual stress level, a resilience psychological questionnaire evaluation can be administered [29, 30, 41]. The neurobiology of resilience is complex [36], but genetic factors are found to play a significant role in resilience [42]. Studies have shown that some psychological attributes connected with resilience could improve with psychotherapeutic interventions [40].

The relationship between corticosteroids and resilience are discussed controversially in the literature. García-León et al. [43] noted an association of hair cortisol with resilience, but without reaching statistical significance. In adolescents there was no statistical significance correlating hair glucocorticoids and the use of a coping strategy known as “shift-persist” [44]. In youth residential caregivers, Bürgin et al. [45] demonstrated a negative correlation of resilience to hair-cortisol/DHEA-ratio.

The overall changes in the concentrations of endocannabinoids and endocannabinoid-like compounds were not high, but indicate a differential pattern. The endocannabinoids/endocannabinoid-like compounds PEA, OEA, SEA and AEA significantly decreased over the observed time course of the pandemic. This decrease was also evident when expressed in their relative values, as compared to the time point reference “January” (which is prior to the pandemic and with less data exclusions than in December). In contrast, 2-AG increased significantly, especially in March and April, and was higher in nurses and workers in the laboratory/ hospital administration group.

2-AG seems to play the most important role of the endocannabinoids [46], as reported in prior experiments involving both animals and humans. Hill et al. [47] described previously in 2009 that levels of 2-AG increased in women undergoing stress, while AEA levels were not affected. Interestingly, an inverse relation between 2-AG and AEA has been described in rats, where stress seems to increase 2-AG, while AEA decreases [17, 48]. Nevertheless, Dlugos et al. [6] could not demonstrate the latter inverse relationship in their study of an acute stress model in healthy subjects. Interestingly, in mice 2-AG increases during stress, but normalizes after removing stress load [49]. Higher 2-AG levels in hair were associated with depressive symptoms in young refugees [15]. In addition, fear-related increases of 2-AG and AEA in rats has been reported [50]. The variability of these responses might be due to the different experimental conditions and cohorts investigated, as reflected by Behnke et al. [51]. He demonstrated a difference of hair cortisol and cortisone levels in women with major depressive disorder in comparison to healthy women, but no difference in the endocannabinoid-levels. Gao et al. [52] did not detect any correlation between stress and hair endocannabinoids while investigating a smaller population.

While several reports on AEA and 2-AG and the relation to stressful conditions are available as described above [19, 48], only few address PEA or SEA in the context of stress. We observed a positive correlation between resilience and PEA concentration, with lower PEA observed in subjects with higher levels of stress.

In regards to SEA, Behnke et al. [14] demonstrated that higher workload correlated with lower SEA, matching our findings that SEA was significantly lower in stressed staff at Visit 3. Wilker et al. [53] noted significantly lower OEA in patients with posttraumatic stress disorder, while we could not find any significant changes in OEA in our study. Because our findings in hair were not confirmed or expanded (e.g. by PEA and SEA serum concentrations), it would be of interest to specifically examine the role of PEA and SEA in a broader study population and including a higher time resolution, especially as one study describes PEA declining during the stress recovery phase [7, 47].

### Strengths and limitations

Hair analyses allows a look back to “normal times”, before the pandemic hit Europe and this study therefore helps investigating and evaluation the role of hair endocannabinoids/ endocannabinoid-like compounds in relation to cortisone and self-evaluating stress questionnaires in the course of the first wave of the COVID-19 pandemic in Germany. However, in this study, the common problem with self-evaluation is to be faced as one cannot assure that questionnaires are without self-bias. Moreover, there are many factors that influence the endocannabinoid- and the cortisone system. Practical limitations of the field study prevented the collecting of data on the subject´s private life or to explore differences in personality. As stated, visits and sample collections happened during the daily work shift. This resulted in some time delay and two timeframes for cutting hair, incurring drop-outs and incomplete data. Also, due to the normal distribution of HCW in our institution, more female than men subjects were enrolled. The statistical strength could have been further increased using a larger population. Practically, the concentration of AEA in hair is very low, so AEA could not be detected in some samples.

To conclude considering all limitations, we could demonstrate in this field study the value of hair analyses of cortisone and endocannabinoids/ endocannabinoid-like compounds as a non-invasive stress marker suite. Lower resilience and higher stress during the pandemic relate to endocannabinoids/ endocannabinoid-like compound concentrations in hair, especially of PEA and 2-AG. A correlation between subjective perceived stress and resilience to cortisone concentrations with a dependency on profession and demographic factors was also observed. Based on these observations and risk assessment, the awareness of increased stress, especially among women, nurses and the elderly, could be considered as an important component of hospital pandemic planning in the future. Therefore, the relevance of monitoring and quantifying psychological health to implement preventive measures and procedures, should be considered useful in protecting and supporting vulnerable groups in coping with these extreme conditions.

### Supplementary information

Supplement

## Data Availability

Data supporting the findings of this study are with the corresponding author.
