# Supplementary material for: Endocannabinoids, endocannabinoid-like compounds and cortisone in head hair of health care workers as markers of stress and resilience during the early COVID-19 pandemic
Source: Transl Psychiatry. 2024 Jan 31;14:71. doi: 10.1038/s41398-024-02771-9 (PMC10831098; doi:10.1038/s41398-024-02771-9)
Supplement: Supplementary file 1 — Supplement [file 41398_2024_2771_MOESM1_ESM.pdf]

Supplement 1: Questionnaire in German (as used) and translated in English

| | Gar nicht | | | | Sehr |
|---|---|---|---|---|---|
| **I. Stress / Angst** | | | | | |
| 1. Wie besorgt sind Sie aktuell, dass Sie an COVID-19 erkranken? | 0 | 1 | 2 | 3 | 4 |
| 2. Wie besorgt waren Sie in den vergangenen 2 Wochen um Ihre Gesundheit aufgrund der COVID-19-Pandemie? | 0 | 1 | 2 | 3 | 4 |
| 3. Wie hoffnungsvoll haben Sie sich in den vergangenen 2 Wochen gefühlt, dass die Corona-Krise bald ein Ende hat? | 0 | 1 | 2 | 3 | 4 |
| 4. Wie sehr haben Sie sich in den vergangenen 2 Wochen aufgrund der COVID-19-Pandemie gestresst bzw. belastet gefühlt? | 0 | 1 | 2 | 3 | 4 |
| **II. Änderung des Verhaltens** | | | | | |
| 1. Wie sehr haben Sie aufgrund der COVID-19-Pandemie in den vergangenen 2 Wochen die empfohlene Maßnahme, Sozialkontakte zu reduzieren, befolgt? | 0 | 1 | 2 | 3 | 4 |
| 2. Wie sehr haben Sie aufgrund der COVID-19-Pandemie in den vergangenen 2 Wochen die empfohlenen Hygienevorschriften befolgt? | 0 | 1 | 2 | 3 | 4 |
| 3. Wie sehr haben Sie aufgrund der COVID-19-Pandemie in den vergangenen 2 Wochen die Maßnahme der Ausgangsbeschränkungen befolgt? | 0 | 1 | 2 | 3 | 4 |
| **III. Auswirkungen der Coronavirus-Pandemie auf die psychische Gesundheit** | | | | | |
| 1. Wie sehr haben Sie aufgrund der COVID-19-Pandemie in den vergangenen 2 Wochen an Ein- und Durchschlafstörungen oder frühmorgendlichem Erwachen gelitten? | 0 | 1 | 2 | 3 | 4 |
| 2. Wie sehr haben Sie aufgrund der COVID-19-Pandemie in den vergangenen 2 Wochen unter Angst- und Paniktattacken mit körperlichen Symptomen (z.B. Herzklopfen, Brustschmerzen, Schwindel) gelitten? | 0 | 1 | 2 | 3 | 4 |
| 3. Wie sehr haben Sie in den vergangenen 2 Wochen mit sich aufdrängenden Bildern, Erinnerungen oder Albträumen über die COVID-19-Pandemie zu kämpfen gehabt? | 0 | 1 | 2 | 3 | 4 |
| 4. Wie sehr haben Sie aufgrund der COVID-19-Pandemie in den vergangenen 2 Wochen einen übermäßig starken Drang verspürt, sich immer wieder die Hände zu waschen und/oder zu desinfizieren, damit Sie nicht von Verschmutzungen oder Keimen krank werden? | 0 | 1 | 2 | 3 | 4 |

| **IV. Umgang mit der Coronavirus-Pandemie** | Gar nicht | | | | Sehr |
|---|---|---|---|---|---|
| 1. Ich neige dazu, mich nach schwierigen Zeiten schnell zu erholen. | 1 | 2 | 3 | 4 | 5 |
| 2. Es fällt mir schwer, stressige Situationen zu überstehen. | 5 | 4 | 3 | 2 | 1 |
| 3. Ich brauche nicht viel Zeit, um mich von einem stressigen Ereignis zu erholen. | 1 | 2 | 3 | 4 | 5 |
| 4. Es fällt mir schwer zur Normalität zurückzukehren, wenn etwas Schlimmes passiert ist. | 5 | 4 | 3 | 2 | 1 |
| 5. Normalerweise überstehe ich schwierige Zeiten ohne größere Probleme. | 1 | 2 | 3 | 4 | 5 |
| 6. Ich brauche tendenziell lange, um über Rückschläge in meinem Leben hinwegzukommen. | 5 | 4 | 3 | 2 | 1 |

| | Not agree | | | | Totally agree |
|---|---|---|---|---|---|
| **I. Stress/anxiety** | | | | | |
| 1. Please indicate how likely you think it is that you will be infected with COVID-19 | 0 | 1 | 2 | 3 | 4 |
| 2. How afraid have you been for the last 14 days for your health because of the COVID-19 pandemic? | 0 | 1 | 2 | 3 | 4 |
| 3. How hopeful have you been the last 14 days that the COVID-19 pandemic is soon over? | 0 | 1 | 2 | 3 | 4 |
| 4. Because of the COVID-19 pandemic I was very stressed over the past 14 days? | 0 | 1 | 2 | 3 | 4 |
| **II. Adhering the rules:** | | | | | |
| 1. Over the last 14 days, I obeyed the reduction of social contacts as recommended? | 0 | 1 | 2 | 3 | 4 |
| 2. Over the last 14 days, I obeyed the hygiene measures as recommended? | 0 | 1 | 2 | 3 | 4 |
| 3. Over the last 14 days, I obeyed the curfew? | 0 | 1 | 2 | 3 | 4 |
| **III. Impact of the psychological health:** | | | | | |
| Over the last 14 days, I suffered from problems, such as difficulty falling asleep, difficulty sleeping through the night or early morning awakening | 0 | 1 | 2 | 3 | 4 |
| Over the last 14 days, I suffered from unforeseeable severe anxiety attacks (panic) with physical symptoms (e.g. palpitations, chest pain, dizziness). | 0 | 1 | 2 | 3 | 4 |
| Over the last 14 days, I suffered from upsetting dreams that replay part of the experience of the COVID-19 pandemic or are clearly related to it | 0 | 1 | 2 | 3 | 4 |
| Over the last 14 days, I suffered from the excessive urge to wash and/or disinfect my hands again and again so that I do not pass on germs or contamination to other people. | 0 | 1 | 2 | 3 | 4 |
| **IV: Resilience** | | | | | |
| 1. I tend to bounce back quickly after hard times | 1 | 2 | 3 | 4 | 5 |
| 2. I have a hard time making it throuh stressful events | 5 | 4 | 3 | 2 | 1 |
| 3. It does not take me long to recover from a stressful event | 1 | 2 | 3 | 4 | 5 |
| 4. It is hard for me to snap back when something bad happens | 5 | 4 | 3 | 2 | 1 |
| 5. I usually come throuh difficult times with little trouble | 1 | 2 | 3 | 4 | 5 |
| 6. I tend to take a long time to get over set-backs in my life | 5 | 4 | 3 | 2 | 1 |

Supplement 1: Questionnaire in original (as used) and with translation (not used and not entire validated)

Questionnaire with question-set I-IV for Visit 2, 3 and 4; for Visit 1 only the question-sets I-III were applied. Question-set I evaluates anxiety. The question 1 in question-set was not eligible as it asked for the momentary state, while all other questions refer to the situation 2 weeks ago and the hair data, accordingly. Question-set II is about adhering to the new COVID-19 rules. Question-set III is about the impact on the psychological health and question-set IV is the brief resilience scale.

Supplement 2: Mass Transition

| Analyt | Mass transition | Internal Standard | Mass transition |
|---|---|---|---|
| AEA | 348.2/62 | AEA-d4 | 352.2/66 |
| 2-AG | 379.2/287.2, 379.2/269.2 | 2AG-d5 | 384.2/287.2 |
| E | 361.2/161.2 | cortisone-d8 | 369.2/169.2 |
| PEA | 300.2/62 | PEA-d4 | 304.2/62 |
| OEA | 326.2/62 | OEA-d4 | 330.2/66 |
| SEA | 328.2/62 | SEA-d3 | 331/62 |

Supplement 2: Mass Transition for the measurement of the hormones

| Supplement 3a: Model of Question I (anxiety) | | | | | | |
|---|---|---|---|---|---|---|
| | Occupation | | Ward | | Covidward | |
| *Predictors* | *Estimates* | *p* | *Estimates* | *p* | *Estimates* | *p* |
| (Intercept) | 7.75<br>(6.79 – 8.72) | **<0.001** | 7.82<br>(6.85 – 8.79) | **<0.001** | 7.79<br>(6.75 – 8.84) | **<0.001** |
| Visit | -0.67<br>(-0.81 – -0.54) | **<0.001** | -0.68<br>(-0.81 – -0.54) | **<0.001** | -0.67<br>(-0.81 – -0.54) | **<0.001** |
| Age-Group: 20 to 29 | *Reference* | | *Reference* | | *Reference* | |
| Age-Group: 30 to 39 | 0.72<br>(-0.12 – 1.56) | 0.093 | 0.72<br>(-0.12 – 1.56) | 0.092 | 0.78<br>(-0.06 – 1.62) | 0.067 |
| Age-Group: 40 to 49 | 1.25<br>(0.30 – 2.20) | **0.010** | 1.19<br>(0.24 – 2.14) | **0.014** | 1.24<br>(0.28 – 2.19) | **0.011** |
| Age-Group: 50 to 59 | 1.51<br>(0.45 – 2.57) | **0.005** | 1.49<br>(0.43 – 2.55) | **0.006** | 1.52<br>(0.45 – 2.59) | **0.006** |
| Age-Group: 60 to 69 | -0.31<br>(-1.83 – 1.21) | 0.690 | -0.32<br>(-1.83 – 1.19) | 0.679 | -0.22<br>(-1.74 – 1.30) | 0.779 |
| Gender: Male | *Reference* | | *Reference* | | *Reference* | |
| Gender: Female | 0.49<br>(-0.27 – 1.26) | 0.206 | 0.50<br>(-0.22 – 1.22) | 0.176 | 0.45<br>(-0.28 – 1.18) | 0.224 |
| Occupation: Physician | *Reference* | | *Reference* | | *Reference* | |
| Occupation: Administration/Laboratory | 0.90<br>(-0.11 – 1.91) | 0.080 | | | | |
| Occupation: Nursing | -0.29<br>(-1.11 – 0.53) | 0.486 | | | | |
| Occupation: Others/Cleaning service | 1.21<br>(-0.33 – 2.75) | 0.125 | | | | |
| Ward: Normal | *Reference* | | *Reference* | | *Reference* | |
| Ward: Administration/Laboratory | | | 0.91<br>(-0.03 – 1.84) | 0.058 | | |
| Ward: Emergency/Endoscopy | | | -0.87<br>(-1.77 – 0.02) | 0.055 | | |
| Ward: ICU | | | 0.21<br>(-0.62 – 1.05) | 0.616 | | |
| Covidward: No | *Reference* | | *Reference* | | *Reference* | |
| Covidward: Yes | | | | | -0.23<br>(-0.99 – 0.52) | 0.544 |
| Covidward: No Patient Contact | | | | | 0.91<br>(-0.06 – 1.87) | 0.065 |
| **Random Effects** | | | | | | |
| $\sigma^2$ | 3.47 | | 3.47 | | 3.47 | |
| $\tau_{00}$ | 3.67 Code | | 3.61 Code | | 3.72 Code | |
| ICC | 0.51 | | 0.51 | | 0.52 | |
| N | 178 Code | | 178 Code | | 178 Code | |
| Observations | 625 | | 625 | | 625 | |
| Marginal $R^2$ / Conditional $R^2$ | 0.151 / 0.587 | | 0.159 / 0.588 | | 0.144 / 0.587 | |

Models of question-set I in dependency of the occupation (column 1), ward (column 2) or COVID-19 ward (column 3). Model of question-set I (stress and anxiety): the age groups 40-49 and 50-59 had a significant higher stress level than than the age group 20-29, the stress level at the ward emergency and endoscopy were significant lower than at the normal ward

| Supplement 3b: Model of Question II (adhering the new COVID-19 rules) | | | | | | |
|---|---|---|---|---|---|---|
| | Occupation | | Ward | | Covidward | |
| *Predictors* | *Estimates* | *p* | *Estimates* | *p* | *Estimates* | *p* |
| (Intercept) | 11.98<br>(11.32 – 12.64) | **<0.001** | 11.73<br>(11.06 – 12.40) | **<0.001** | 11.69<br>(10.97 – 12.40) | **<0.001** |
| Visit | -0.73<br>(-0.83 – -0.63) | **<0.001** | -0.73<br>(-0.83 – -0.63) | **<0.001** | -0.73<br>(-0.83 – -0.62) | **<0.001** |
| Age-Group: 20 to 29 | *Reference* | | *Reference* | | *Reference* | |
| Age-Group: 30 to 39 | 0.36<br>(-0.20 – 0.92) | 0.210 | 0.37<br>(-0.20 – 0.94) | 0.198 | 0.41<br>(-0.16 – 0.97) | 0.158 |
| Age-Group: 40 to 49 | 0.58<br>(-0.06 – 1.21) | 0.076 | 0.55<br>(-0.09 – 1.20) | 0.093 | 0.57<br>(-0.07 – 1.22) | 0.082 |
| Age-Group: 50 to 59 | 0.66<br>(-0.05 – 1.38) | 0.069 | 0.57<br>(-0.16 – 1.30) | 0.123 | 0.59<br>(-0.14 – 1.31) | 0.112 |
| Age-Group: 60 to 69 | 1.51<br>(0.48 – 2.54) | **0.004** | 1.25<br>(0.21– 2.29) | **0.018** | 1.29<br>(0.26 – 2.32) | **0.014** |
| Gender: Male | *Reference* | | *Reference* | | *Reference* | |
| Gender: Female | 0.02<br>(-0.50 – 0.53) | 0.946 | -0.16<br>(-0.65 – 0.34) | 0.534 | -0.17<br>(-0.66 – 0.33) | 0.510 |
| Occupation: Physician | *Reference* | | *Reference* | | *Reference* | |
| Occupation: Administration/Laboratory | 0.01<br>(-0.67 – 0.68) | 0.988 | | | | |
| Occupation: Nursing | -0.57<br>(-1.12 – -0.01) | **0.044** | | | | |
| Occupation: Others/Cleaning service | -0.96<br>(-2.00 – 0.08) | 0.069 | | | | |
| Ward: Normal | *Reference* | | *Reference* | | *Reference* | |
| Ward: Administration/Laboratory | | | 0.48<br>(-0.16 – 1.12) | 0.139 | | |
| Ward: Emergency/Endoscopy | | | -0.26<br>(-0.87 – 0.35) | 0.402 | | |
| Ward: ICU | | | 0.14<br>(-0.44 – 0.71) | 0.638 | | |
| Covidward: No | *Reference* | | *Reference* | | *Reference* | |
| Covidward: Yes | | | | | 0.01<br>(-0.50 – 0.52) | 0.978 |
| Covidward: No Patient Contact | | | | | 0.51<br>(-0.14 – 1.16) | 0.127 |
| **Random Effects** | | | | | | |
| $\sigma^2$ | 2.04 | | 2.04 | | 2.04 | |
| $\tau_{00}$ | 1.53 Code | | 1.56 Code | | 1.57 Code | |
| ICC | 0.43 | | 0.43 | | 0.43 | |
| N | 178 Code | | 178 Code | | 178 Code | |
| Observations | 630 | | 630 | | 630 | |
| Marginal $R^2$ / Conditional $R^2$ | 0.193 / 0.539 | | 0.186 / 0.539 | | 0.184 / 0.538 | |

Models of question-set II in dependency of the occupation (column 1), ward (column 2) or COVID-19 ward (column 3).

Model of question-set II (adhering the new COVID-19 rules): adherence to the new COVID-19 rules was significantly lower in nurses than in physicians and significant higher in the age group 60-69

| Supplement 3c: Model of Question III (psychological health) | | | | | | |
|---|---|---|---|---|---|---|
| | Occupation | | Ward | | Covidward | |
| *Predictors* | *Estimates* | *p* | *Estimates* | *p* | *Estimates* | *p* |
| (Intercept) | 2.20<br>(1.25 – 3.16) | **<0.001** | 2.53<br>(1.58 – 3.48) | **<0.001** | 2.43<br>(1.39 – 3.47) | **<0.001** |
| Visit | -0.39<br>(-0.51 – -0.26) | **<0.001** | -0.40<br>(-0.52 – -0.27) | **<0.001** | -0.39<br>(-0.51 – -0.26) | **<0.001** |
| Age-Group: 20 to 29 | *Reference* | | *Reference* | | *Reference* | |
| Age-Group: 30 to 39 | 0.47<br>(-0.37 – 1.31) | 0.271 | 0.29<br>(-0.53 – 1.11) | 0.490 | 0.45<br>(-0.39 – 1.29) | 0.296 |
| Age-Group: 40 to 49 | 0.60<br>(-0.35 – 1.55) | 0.216 | 0.51<br>(-0.42 – 1.44) | 0.286 | 0.59<br>(-0.37 – 1.55) | 0.225 |
| Age-Group: 50 to 59 | 1.58<br>(0.52 – 2.65) | **0.004** | 1.55<br>(0.50 – 2.59) | **0.004** | 1.63<br>(0.55 – 2.71) | **0.003** |
| Age-Group: 60 to 69 | -0.27<br>(-1.80 – 1.26) | 0.732 | -0.31<br>(-1.80 – 1.18) | 0.683 | -0.10<br>(-1.63 – 1.43) | 0.896 |
| Gender: Male | *Reference* | | *Reference* | | *Reference* | |
| Gender: Female | 0.80<br>(0.03 – 1.57) | **0.041** | 1.07<br>(0.36 – 1.78) | **0.003** | 1.02<br>(0.28 – 1.75) | **0.007** |
| Occupation: Physician | *Reference* | | *Reference* | | *Reference* | |
| Occupation: Administration/Laboratory | 0.83<br>(-0.18 – 1.85) | 0.107 | | | | |
| Occupation: Nursing | 0.55<br>(-0.27 – 1.37) | 0.191 | | | | |
| Occupation: Others/Cleaning service | 1.25<br>(-0.29 – 2.80) | 0.112 | | | | |
| Ward: Normal | *Reference* | | *Reference* | | *Reference* | |
| Ward: Administration/Laboratory | | | 0.38<br>(-0.54 – 1.31) | 0.415 | | |
| Ward: Emergency/Endoscopy | | | -0.91<br>(-1.79 – -0.04) | **0.042** | | |
| Ward: ICU | | | 0.72<br>(-0.10 – 1.55) | 0.085 | | |
| Covidward: No | *Reference* | | *Reference* | | *Reference* | |
| Covidward: Yes | | | | | 0.05<br>(-0.71 – 0.81) | 0.887 |
| Covidward: No Patient Contact | | | | | 0.40<br>(-0.57 – 1.37) | 0.419 |
| **Random Effects** | | | | | | |
| $\sigma^2$ | 2.99 | | 2.99 | | 2.99 | |
| $\tau_{00}$ | 3.86 $_{Code}$ | | 3.61 $_{Code}$ | | 3.92 $_{Code}$ | |
| ICC | 0.56 | | 0.55 | | 0.57 | |
| N | 178 $_{Code}$ | | 178 $_{Code}$ | | 178 $_{Code}$ | |
| Observations | 631 | | 631 | | 631 | |
| Marginal $R^2$ / Conditional $R^2$ | 0.111 / 0.611 | | 0.140 / 0.610 | | 0.101 / 0.611 | |

Models of question-set III in dependency of the occupation (column 1), ward (column 2) or COVID-19 ward (column 3).

Model of question-set III (impact of the psychological health): the impact was significantly higher for females than males and in the age group 50-59, but significantly lower in the emergency ward and endoscopy personnel.

| Supplement 3d: Model of Question IV (resilience) | | | | | | |
|---|---|---|---|---|---|---|
| | **Occupation** | | **Ward** | | **Covidward** | |
| *Predictors* | *Estimates* | *p* | *Estimates* | *p* | *Estimates* | *p* |
| (Intercept) | 24.54 (22.90 – 26.18) | **<0.001** | 24.04 (22.36 – 25.73) | **<0.001** | 23.43 (21.65 – 25.21) | **<0.001** |
| Visit | 0.04 (-0.19 – 0.27) | 0.746 | 0.04 (-0.19 – 0.27) | 0.732 | 0.04 (-0.19 – 0.27) | 0.730 |
| Age-Group: 20 to 29 | *Reference* | | *Reference* | | *Reference* | |
| Age-Group: 30 to 39 | -0.08 (-1.55 – 1.40) | 0.920 | -0.04 (-1.52 – 1.45) | 0.962 | 0.01 (-1.44 – 1.47) | 0.985 |
| Age-Group: 40 to 49 | -0.36 (-2.03 – 1.30) | 0.667 | -0.38 (-2.06 – 1.30) | 0.654 | -0.43 (-2.09 – 1.23) | 0.611 |
| Age-Group: 50 to 59 | -1.44 (-3.31– 0.43) | 0.131 | -1.47 (-3.37 – 0.43) | 0.129 | -1.48 (-3.35 – 0.39) | 0.121 |
| Age-Group: 60 to 69 | 0.66 (-1.99 – 3.31) | 0.623 | 0.58 (-2.07 – 3.24) | 0.666 | 0.60 (-2.02 – 3.22) | 0.654 |
| Gender: Male | *Reference* | | *Reference* | | *Reference* | |
| Gender: Female | -2.03 (-3.38 – -0.69) | **0.003** | -2.38 (-3.66 – -1.11) | **<0.001** | -2.29 (-3.56 – -1.02) | **<0.001** |
| Occupation: Physician | *Reference* | | *Reference* | | *Reference* | |
| Occupation: Administration/Laboratory | -2.08 (-3.86 – -0.31) | **0.022** | | | | |
| Occupation: Nursing | -0.98 (-2.41– 0.46) | 0.181 | | | | |
| Occupation: Others/Cleaning service | -1.30 (-4.00 – 1.39) | 0.343 | | | | |
| Ward: Normal | *Reference* | | *Reference* | | *Reference* | |
| Ward: Administration/Laboratory | | | -1.27 (-2.94 – 0.40) | 0.136 | | |
| Ward: Emergency/Endoscopy | | | 0.10 (-1.49 – 1.69) | 0.903 | | |
| Ward: ICU | | | 0.13 (-1.35 – 1.61) | 0.865 | | |
| Covidward: No | *Reference* | | *Reference* | | *Reference* | |
| Covidward: Yes | | | | | 0.94 (-0.38 – 2.25) | 0.161 |
| Covidward: No Patient Contact | | | | | -0.74 (-2.42 – 0.95) | 0.390 |
| **Random Effects** | | | | | | |
| $\sigma^2$ | 4.31 | | 4.32 | | 4.32 | |
| $\tau_{00}$ | 12.79 $_{Code}$ | | 12.94 $_{Code}$ | | 12.68 $_{Code}$ | |
| ICC | 0.75 | | 0.75 | | 0.75 | |
| N | 178 $_{Code}$ | | 178 $_{Code}$ | | 178 $_{Code}$ | |
| Observations | 488 | | 488 | | 488 | |
| Marginal $R^2$ / Conditional $R^2$ | 0.114 / 0.777 | | 0.105 / 0.776 | | 0.113 / 0.775 | |

Models of question-set IV in dependency of the occupation (column 1), ward (column 2) or COVID-19 ward (column 3).

Model of question-set IV (resilience): the resilience was significantly lower in women than in men and in the occupation administration/laboratory workers than the physicians.

Supplement 4a: Linear Model at Visit 1 with Factorized Stress Score

| | **PEA** | | **OEA** | | **SEA** | | **2-AG** | | **AEA** | |
|---|---|---|---|---|---|---|---|---|---|---|
| *Predictors* | *Estimates* | *p* | *Estimates* | *p* | *Estimates* | *p* | *Estimates* | *p* | *Estimates* | *p* |
| (Intercept) | 7.83 (6.58–9.08) | **<0.001** | 8.25 (6.53–9.97) | **<0.001** | 7.93 (7.02–8.84) | **<0.001** | 4.10 (3.24–4.96) | **<0.001** | 0.28 (-1.11–1.66) | 0.692 |
| Age-Group: 20 to 29 | *Reference* | | *Reference* | | *Reference* | | *Reference* | | *Reference* | |
| Age-Group: 30 to 39 | -0.04 (-0.31 – 0.22) | 0.757 | 0.00 (-0.36 – 0.36) | 0.996 | -0.06 (-0.26 – 0.13) | 0.510 | -0.06 (-0.25 – 0.12) | 0.481 | -0.18 (-0.49 – 0.14) | 0.268 |
| Age-Group: 40 to 49 | -0.01 (-0.32 – 0.30) | 0.949 | -0.02 (-0.45 – 0.40) | 0.923 | -0.07 (-0.30 – 0.16) | 0.540 | 0.00 (-0.21 – 0.22) | 0.978 | -0.01 (-0.40 – 0.38) | 0.962 |
| Age-Group: 50 to 59 | -0.13 (-0.46 – 0.21) | 0.461 | -0.18 (-0.64 – 0.28) | 0.444 | -0.03 (-0.27 – 0.22) | 0.834 | 0.00 (-0.23 – 0.23) | 0.988 | -0.01 (-0.40 – 0.38) | 0.965 |
| Age-Group: 60 to 69 | -0.33 (-0.88 – 0.23) | 0.245 | -0.14 (-0.90 – 0.62) | 0.719 | -0.18 (-0.58 – 0.22) | 0.373 | 0.06 (-0.32 – 0.44) | 0.755 | -0.01 (-0.76 – 0.73) | 0.972 |
| Gender: Male | *Reference* | | *Reference* | | *Reference* | | *Reference* | | *Reference* | |
| Gender: Female | 0.10 (-0.15 – 0.36) | 0.431 | 0.04 (-0.31 – 0.40) | 0.802 | -0.08 (-0.26 – 0.11) | 0.402 | -0.08 (-0.26 – 0.09) | 0.349 | -0.01 (-0.35 – 0.33) | 0.954 |
| Occupation: Physician | *Reference* | | *Reference* | | *Reference* | | *Reference* | | *Reference* | |
| Occupation: Administration/Laboratory | 0.13 (-0.21 – 0.47) | 0.440 | 0.30 (-0.16 – 0.76) | 0.204 | 0.06 (-0.18 – 0.31) | 0.599 | 0.36 (0.13 – 0.59) | **0.002** | 0.03 (-0.37 – 0.43) | 0.881 |
| Occupation: Nursing | -0.01 (-0.28 – 0.25) | 0.917 | -0.02 (-0.38 – 0.35) | 0.930 | -0.10 (-0.29 – 0.09) | 0.312 | 0.26 (0.08 – 0.44) | **0.005** | 0.25 (-0.07 – 0.57) | 0.118 |
| Occupation: Others/Cleaning service | -0.23 (-0.87 – 0.40) | 0.468 | -0.24 (-1.11 – 0.64) | 0.592 | -0.08 (-0.54 – 0.38) | 0.736 | 0.15 (-0.24 – 0.54) | 0.458 | -0.45 (-1.23 – 0.33) | 0.253 |
| CovidContact: No | *Reference* | | *Reference* | | *Reference* | | *Reference* | | *Reference* | |
| CovidContact: Yes | -0.00 (-0.24 – 0.24) | 0.998 | 0.00 (-0.32 – 0.33) | 0.990 | -0.03 (-0.20 – 0.14) | 0.747 | 0.08 (-0.08 – 0.24) | 0.330 | -0.13 (-0.41 – 0.15) | 0.372 |
| Visit 1 Stress / Anxiety: low stress | *Reference* | | *Reference* | | *Reference* | | *Reference* | | *Reference* | |
| Visit 1 Stress / Anxiety: Stressed | -0.21 (-0.44 – 0.03) | 0.090 | -0.22 (-0.54 – 0.11) | 0.189 | -0.23 (-0.40 – -0.06) | **0.010** | -0.08 (-0.24 – 0.08) | 0.320 | -0.13 (-0.43 – 0.16) | 0.366 |
| Visit 1 Adhering rules: Not Changed | *Reference* | | *Reference* | | *Reference* | | *Reference* | | *Reference* | |
| Visit 1 adhering rules: Changed | 0.02 (-1.14 – 1.18) | 0.975 | -0.85 (-2.44 – 0.74) | 0.292 | 0.13 (-0.71 – 0.97) | 0.758 | 0.67 (-0.12 – 1.47) | 0.096 | 0.19 (-1.07 – 1.44) | 0.769 |
| Visit 1 Effects of the Pandemic: Not Impacted | *Reference* | | *Reference* | | *Reference* | | *Reference* | | *Reference* | |
| Visit 1 Effects of the Pandemic: Impacted | 0.37 (-0.08 – 0.83) | 0.107 | 0.27 (-0.35 – 0.90) | 0.385 | 0.04 (-0.31 – 0.39) | 0.819 | -0.10 (-0.41 – 0.21) | 0.536 | -0.18 (-0.71 – 0.35) | 0.512 |
| Observations | 122 | | 122 | | 122 | | 123 | | 99 | |
| $R^2$ / $R^2$ adjusted | 0.099 / -0.019 | | 0.069 / -0.053 | | 0.108 / -0.009 | | 0.148 / 0.038 | | 0.117 / -0.030 | |

Supplement 4a: Linear Model at Visit 1. The occupation nursing and Laboratory/administration have higher 2-AG level at Visit 1 than physicians.

Supplement 4b: Linear Model at Visit 3 with Factorized Stress Score

| | **PEA** | | **OEA** | | **SEA** | | **2-AG** | | **AEA** | |
|---|---|---|---|---|---|---|---|---|---|---|
| *Predictors* | *Estimates* | *p* | *Estimates* | *p* | *Estimates* | *p* | *Estimates* | *p* | *Estimates* | *p* |
| (Intercept) | 7.62 (7.24 – 8.01) | **<0.001** | 7,24 (6.76 – 7.72) | **<0.001** | 7.61 (7.26 – 7.95) | **<0.001** | 4.88 (4.63 – 5.13) | **<0.001** | 0.06 (-0.48 – 0.60) | 0.833 |
| Age-Group: 20 to 29 | *Reference* | | *Reference* | | *Reference* | | *Reference* | | *Reference* | |
| Age-Group: 30 to 39 | 0.06 (-0.15 – 0.28) | 0.569 | 0.06 (-0.21 – 0.33) | 0.661 | 0.03 (-0.16 – 0.22) | 0.776 | -0.03 (-0.16 – 0.11) | 0.718 | -0.04 (-0.35 – 0.27) | 0.791 |
| Age-Group: 40 to 49 | -0.03 (-0.27 – 0.21) | 0.825 | 0.02 (-0.28 – 0.32) | 0.902 | -0.05 (-0.26 – 0.16) | 0.645 | 0.01 (-0.15 – 0.16) | 0.941 | 0.04 (-0.33 – 0.40) | 0.847 |
| Age-Group: 50 to 59 | 0.09 (-0.19 – 0.38) | 0.522 | 0.13 (-0.22 – 0.49) | 0.464 | 0.00 (-0.26 – 0.26) | 0.978 | 0.06 (-0.13 – 0.24) | 0.547 | 0.01 (-0.39 – 0.41) | 0.959 |
| Age-Group: 60 to 69 | -0.17 (-0.58 – 0.23) | 0.395 | 0.08 (-0.42 – 0.58) | 0.756 | -0.05 (-0.41 – 0.31) | 0.787 | 0.28 (0.02 – 0.54) | **0.035** | 0.22 (-0.45 – 0.89) | 0.519 |
| Gender: Male | *Reference* | | *Reference* | | *Reference* | | *Reference* | | *Reference* | |
| Gender: Female | 0.16 (-0.04 – 0.36) | 0.116 | 0.03 (-0.22 – 0.28) | 0.807 | 0.02 (-0.16 – 0.20) | 0.828 | -0.06 (-0.19 – 0.07) | 0.332 | 0.06 (-0.25 – 0.37) | 0.711 |
| Occupation: Physician | *Reference* | | *Reference* | | *Reference* | | *Reference* | | *Reference* | |
| Occupation: Administration/Laboratory | 0.20 (-0.07 – 0.47) | 0.141 | 0.21 (-0.13 – 0.54) | 0.224 | 0.23 (-0.01 – 0.47) | 0.062 | 0.29 (0.11 – 0.46) | **0.001** | -0.04 (-0.42 – 0.35) | 0.845 |
| Occupation: Nursing | 0.01 (-0.20 – 0.23) | 0.904 | 0.03 (-0.24 – 0.29) | 0.836 | 0.03 (-0.16 – 0.22) | 0.761 | 0.22 (0.08 – 0.36) | **0.002** | 0.21 (-0.11 – 0.52) | 0.191 |
| Occupation: Others/Cleaning service | -0.01 (-0.42 – 0.40) | 0.977 | 0.09 (-0.42 – 0.60) | 0.721 | 0.11 (-0.26 – 0.47) | 0.571 | 0.20 (-0.05 – 0.45) | 0.119 | 0.21 (-0.39 – 0.81) | 0.489 |
| CovidContact: No | *Reference* | | *Reference* | | *Reference* | | *Reference* | | *Reference* | |
| CovidContact: Yes | 0.10 (-0.09 – 0.28) | 0.296 | 0.08 (-0.15 – 0.30) | 0.517 | 0.16 (-0.01 – 0.32) | 0.062 | 0.12 (0.00 – 0.24) | **0.044** | -0.11 (-0.39 – 0.16) | 0.418 |
| Visit 3 Stress / Anxiety: low stress | *Reference* | | *Reference* | | *Reference* | | *Reference* | | *Reference* | |
| Visit 3 Stress / Anxiety: Stressed | -0.26 (-0.49 – -0.03) | **0.026** | -0.17 (-0.45 – 0.12) | 0.246 | -0.12 (-0.33 – 0.08) | 0.235 | -0.08 (-0.23 – 0.07) | 0.304 | 0.01 (-0.33 – 0.35) | 0.963 |
| Visit 3 Adhering rules: Not Changed | *Reference* | | *Reference* | | *Reference* | | *Reference* | | *Reference* | |
| Visit 3 Adhering rules: Changed | -0.02 (-0.26 – 0.22) | 0.878 | 0.05 (-0.26 – 0.35) | 0.763 | 0.06 (-0.15 – 0.28) | 0.560 | -0.00 (-0.16 – 0.15) | 0.967 | 0.05 (-0.29 – 0.40) | 0.754 |
| Visit 3 Effects of the Pandemic: Not Impacted | *Reference* | | *Reference* | | *Reference* | | *Reference* | | *Reference* | |
| Visit 3 Effects of the Pandemic: Impacted | 0.68 (-0.37 – 1.73) | 0.203 | 0.95 (-0.36 – 2.25) | 0.153 | 0.40 (-0.53 – 1.34) | 0.396 | -0.17 (-0.85 – 0.51) | 0.618 | -0.16 (-1.53 – 1.21) | 0.815 |
| Visit 3 Resilience: Low resilience | *Reference* | | *Reference* | | *Reference* | | *Reference* | | *Reference* | |
| Visit 3 Resilience: Normal resilience | 0.16 (-0.09 – 0.41) | 0.202 | -0.00 (-0.31 – 0.31) | 0.988 | 0.08 (-0.14 – 0.30) | 0.494 | -0.13 (-0.30 – 0.03) | 0.108 | 0.05 (-0.29 – 0.39) | 0.769 |
| Visit 3 Resilience: High resilience | 0.37 (0.07 – 0.66) | **0.014** | 0.27 (-0.09 – 0.64) | 0.142 | 0.20 (-0.06 – 0.46) | 0.130 | -0.10 (-0.29 – 0.09) | 0.317 | 0.27 (-0.14 – 0.68) | 0.190 |
| Observations | 156 | | 155 | | 155 | | 156 | | 126 | |
| $R^2$ / $R^2$ adjusted | 0.140 / 0.054 | | 0.079 / -0.013 | | 0.089 / -0.002 | | 0.171 / 0.089 | | 0.071 / -0.047 | |

Supplement 4: Linear Model at Visit 3. The occupation nursing and Laboratory/administration have higher 2-AG level at Visit 3.
There are also higher 2-AG-levels with subjects with COVID-19 contact. Regarding PEA, significantly higher PEA-levels in subjects with higher resilience was seen, while lower PEA-concentrations were quantified in stressed subjects.

Supplement 5: Mixed Model without Hair-related Factors

| **Supplement 5: Mixed Model without Hair-related Factors** | | | | | | | | | | |
|---|---|---|---|---|---|---|---|---|---|---|
| | **PEA** | | **OEA** | | **SEA** | | **2-AG** | | **AEA** | |
| *Predictors* | *Estimates* | *p* | *Estimates* | *p* | *Estimates* | *p* | *Estimates* | *p* | *Estimates* | *p* |
| (Intercept) | 8.16 (7.84 – 8.48) | **<0.001** | 7.79 (7.33 – 8.25) | **<0.001** | 8.07 (7.85 – 8.29) | **<0.001** | 4.62 (4.43 – 4.80) | **<0.001** | 0.37 (0.05 – 0.69) | **0.024** |
| Age-Group: 20 to 29 | *Reference* | | *Reference* | | *Reference* | | *Reference* | | *Reference* | |
| Age-Group: 30 to 39 | 0.06 (-0.19–0.32) | 0.626 | 0.07 (-0.29–0.43) | 0.713 | 0.01 (-0.16–0.17) | 0.945 | -0.08 (-0.22–0.07) | 0.297 | -0.10 (-0.34–0.14) | 0.403 |
| Age-Group: 40 to 49 | -0.05 (-0.34–0.23) | 0.721 | -0.11 (-0.52–0.30) | 0.589 | -0.12 (-0.31–0.07) | 0.225 | -0.09 (-0.25–0.08) | 0.303 | 0.09 (-0.19–0.38) | 0.525 |
| Age-Group: 50 to 59 | -0.02 (-0.33–0.30) | 0.904 | -0.07 (-0.53–0.38) | 0.750 | -0.04 (-0.25–0.17) | 0.722 | -0.08 (-0.26–0.10) | 0.379 | -0.07 (-0.37–0.23) | 0.641 |
| Age-Group: 60 to 69 | -0.29 (-0.73–0.16) | 0.206 | -0.36 (-1.00–0.28) | 0.275 | -0.12 (-0.42–0.17) | 0.411 | 0.18 (-0.08–0.43) | 0.173 | 0.07 (-0.37–0.50) | 0.756 |
| Gender: Male | *Reference* | | *Reference* | | *Reference* | | *Reference* | | *Reference* | |
| Gender: Female | 0.04 (-0.19–0.28) | 0.729 | 0.13 (-0.21–0.46) | 0.455 | -0.04 (-0.20–0.11) | 0.593 | 0.01 (-0.12–0.15) | 0.838 | 0.06 (-0.18–0.29) | 0.639 |
| Occupation: Physician | *Reference* | | *Reference* | | *Reference* | | *Reference* | | *Reference* | |
| Occupation: Administration/Laboratory | 0.21 (-0.11–0.53) | 0.192 | 0.19 (-0.26–0.65) | 0.408 | 0.13 (-0.08–0.35) | 0.231 | 0.20 (0.01–0.39) | **0.037** | 0.00 (-0.31–0.31) | 0.997 |
| Occupation: Nursing | 0.05 (-0.20–0.31) | 0.671 | -0.08 (-0.44–0.27) | 0.640 | -0.00 (-0.17–0.16) | 0.995 | 0.16 (0.02–0.30) | **0.027** | 0.31 (0.07–0.55) | **0.013** |
| Occupation: Others/Cleaning service | -0.01 (-0.49–0.46) | 0.951 | -0.12 (-0.80–0.55) | 0.719 | -0.02 (-0.33–0.29) | 0.905 | 0.21 (-0.04–0.47) | 0.103 | -0.04 (-0.49–0.42) | 0.876 |
| CovidContact: No | *Reference* | | *Reference* | | *Reference* | | *Reference* | | *Reference* | |
| CovidContact: Yes | 0.11 (-0.10–0.33) | 0.297 | 0.15 (-0.15–0.46) | 0.328 | 0.07 (-0.07–0.21) | 0.337 | 0.17 (0.05–0.29) | **0.005** | -0.04 (-0.25–0.16) | 0.690 |
| Seroconversion: No | *Reference* | | *Reference* | | *Reference* | | *Reference* | | *Reference* | |
| Seroconversion: Yes | -0.12 (-0.53–0.29) | 0.561 | -0.12 (-0.71–0.46) | 0.685 | -0.18 (-0.46–0.09) | 0.184 | -0.16 (-0.40–0.07) | 0.168 | 0.04 (-0.36–0.45) | 0.832 |
| Time | -0.06 (-0.07– -0.04) | **<0.001** | -0.07 (-0.09– -0.06) | **<0.001** | -0.03 (-0.05– -0.02) | **<0.001** | 0.03 (0.01–0.04) | **<0.001** | -0.04 (-0.06– -0.02) | **0.001** |
| **Random Effects** | | | | | | | | | | |
| $\sigma^2$ | 0.08 | | 0.13 | | 0.10 | | 0.07 | | 0.15 | |
| $\tau_{00}$ | 0.39 $_{Subject}$ | | 0.81 $_{Subject}$ | | 0.16 $_{Subject}$ | | 0.12 $_{Subject}$ | | 0.29 $_{Subject}$ | |
| ICC | 0.84 | | 0.86 | | 0.61 | | 0.64 | | 0.66 | |
| N | 171 $_{Subject}$ | | 174 $_{Subject}$ | | 171 $_{Subject}$ | | 174 $_{Subject}$ | | 153 $_{Subject}$ | |
| Observations | 823 | | 837 | | 822 | | 832 | | 638 | |
| Marginal $R^2$ / Conditional $R^2$ | 0.042 / 0.843 | | 0.038 / 0.867 | | 0.037 / 0.624 | | 0.100 / 0.680 | | 0.086 / 0.689 | |

Supplement 5: Mixed Model without hair related factors over the whole time period

A significant higher concentration of 2-AG in nurses and administration/laboratory workers was quantified. The decrease of PEA, OEA, SEA and AEA and an increase of 2-AG over the time-period was significant.

Supplement 6: Linear Model of cortisone at Visit 1 (6a) and Visit 3 (6b) with Factorized Stress Score

**Supplement 6a: Linear Model of Cortisone at Visit 1 with Factorized Stress Score**

| | Cortisone | |
|---|---|---|
| *Predictors* | *Estimates* | *p* |
| (Intercept) | 16.00 (-16.53 – 48.52) | 0.332 |
| Age-Group: 20 to 29 | *Reference* | |
| Age-Group: 30 to 39 | -2.94 (-10.08 – 4.19) | 0.415 |
| Age-Group: 40 to 49 | 3.56 (-4.39 – 11.52) | 0.376 |
| Age-Group: 50 to 59 | -0.58 (-9.29 – 8.13) | 0.894 |
| Age-Group: 60 to 69 | -0.58 (-14.73 – 13.58) | 0.936 |
| Gender: Male | *Reference* | |
| Gender: Female | -5.33 (-12.21 – 1.54) | 0.127 |
| Occupation: Physician | *Reference* | |
| Occupation: Administration/Laboratory | 1.00 (-7.97 – 9.96) | 0.826 |
| Occupation: Nursing | 9.50 (2.31 – 16.70) | **0.010** |
| Occupation: Others/Cleaning service | -5.02 (-21.06 – 11.02) | 0.536 |
| CovidContact: No | *Reference* | |
| CovidContact: Yes | -4.53 (-10.66 – 1.61) | 0.146 |
| Seroconversion: No | *Reference* | |
| Seroconversion: Yes | 0.38 (-17.48 – 18.23) | 0.967 |
| Block I: Stress / Anxiety: Not Stressed | *Reference* | |
| Block I: Stress / Anxiety: Stressed | 4.44 (-1.83 – 10.71) | 0.163 |
| Block II: Adhering Rules: Not Changed | *Reference* | |
| Block II: Adhering Rules: Changed | 13.02 (-16.49 – 42.53) | 0.383 |
| Block III: Effects of the Pandemic: Not Impacted | *Reference* | |
| Block III: Effects of the Pandemic: Impacted | -7.43 (-19.90 – 5.03) | 0.239 |
| Block IV: Resilience: Low resilience | *Reference* | |
| Block IV: Resilience: Normal resilience | 0.25 (-8.64 – 9.13) | 0.956 |
| Block IV: Resilience: High resilience | -3.21 (-13.71 – 7.29) | 0.545 |
| Observations | 116 | |
| $R^2$ / $R^2$ adjusted | 0.176 / 0.052 | |

**Supplement 6b: Linear Model of Cortisone at Visit 3 with Factorized Stress Score**

| | Cortisone | |
|---|---|---|
| *Predictors* | *Estimates* | *p* |
| (Intercept) | 43.26 (29.65 – 56.87) | **<0.001** |
| Age-Group: 20 to 29 | *Reference* | |
| Age-Group: 30 to 39 | -1.49 (-9.24 – 6.26) | 0.704 |
| Age-Group: 40 to 49 | 8.92 (0.46 – 17.38) | **0.039** |
| Age-Group: 50 to 59 | 6.15 (-3.91 – 16.21) | 0.229 |
| Age-Group: 60 to 69 | 3.48 (-12.34 – 19.29) | 0.664 |
| Gender: Male | *Reference* | |
| Gender: Female | -9.09 (-16.33 – -1.85) | **0.014** |
| Occupation: Physician | *Reference* | |
| Occupation: Administration/Laboratory | 8.01 (-1.79 – 17.81) | 0.108 |
| Occupation: Nursing | 10.82 (3.01 – 18.64) | **0.007** |
| Occupation: Others/Cleaning service | 6.12 (-9.92 – 22.15) | 0.452 |
| CovidContact: No | *Reference* | |
| CovidContact: Yes | 1.33 (-5.27 – 7.93) | 0.691 |
| Seroconversion: No | *Reference* | |
| Seroconversion: Yes | 4.21 (-9.41 – 17.83) | 0.542 |
| Block I: Stress / Anxiety: Not Stressed | *Reference* | |
| Block I: Stress / Anxiety: Stressed | 5.08 (-3.02 – 13.17) | 0.217 |
| Block II: Adhering Rules: Not Changed | *Reference* | |
| Block II: Adhering Rules: Changed | -3.91 (-12.37 – 4.54) | 0.362 |
| Block III: Effects of the Pandemic: Not Impacted | *Reference* | |
| Block III: Effects of the Pandemic: Impacted | -13.46 (-49.58 – 22.67) | 0.462 |
| Block IV: Resilience: Low resilience | *Reference* | |
| Block IV: Resilience: Normal resilience | -7.43 (-16.09 – 1.23) | 0.092 |
| Block IV: Resilience: High resilience | -7.41 (-17.72 – 2.91) | 0.158 |
| Observations | 148 | |
| $R^2$ / $R^2$ adjusted | 0.137 / 0.039 | |

Supplement 6: Linear Model of cortisone at Visit 1 (6a) and Visit 3 (6b) with Factorized Stress Score At both visits, nurses had higher cortisone levels than physicians. At Visit 3 significant higher cortisone level in the age group 40-49 than in the age group 20-29 and significant lower cortisone level in female were observed.
